# Urbanization-driven environmental shifts cause reduction in aminopeptidase N activity in the honeybee

**DOI:** 10.1093/conphys/coae073

**Published:** 2024-12-12

**Authors:** Andrea Ferrari, Silvia Caccia, Carlo Polidori

**Affiliations:** Department of Environmental Science and Policy (ESP), University of Milan, via Celoria 26, 20133, Milan, Italy; Department of Biosciences, University of Milan, via Celoria 26, Milan 20133, Italy; Department of Environmental Science and Policy (ESP), University of Milan, via Celoria 26, 20133, Milan, Italy

**Keywords:** Aminopeptidase n, honeybees, insect midgut, land use change, urbanization

## Abstract

Honeybees (*Apis mellifera* Linnaeus, 1758) are managed pollinators in anthropized landscapes but suffer adverse physiological effects from urbanization due to increased pollution, higher temperatures and a loss of habitat quality. Previous studies in various animal taxa have shown how responses of digestive enzymes, such as Aminopeptidase N (APN), can indicate stress conditions and thus be used to measure the harmfulness of anthropogenic disturbance. However, no studies have focused on bees. Here, we sampled honeybee foragers along an urbanization gradient in the Metropolitan City of Milan (Italy) and measured the APN activity. After briefly characterizing the midgut APN activity under different pH and temperature conditions, we found that APN activity was lower at urban sites with higher temperatures (Urban Heat Island (UHI) effect). Furthermore, an increasing proportion of meadows (semi-natural flowered areas) and a decreasing proportion of urban parks (managed urban green areas)—both higher in less urbanized sites—were associated with higher APN activity. Our results suggest that severe urban conditions may cause a reduction in APN activity, but that the UHI effect alone is not directly involved. Although the actual urbanization-related factors driving our results remain unclear, we suggest that impoverishment of food sources may play a role. As aminopeptidases are involved in pollen digestion, our results may indicate a possible impairment of the digestive capacity of honeybees in highly urbanized areas.

## Abbreviations

Apn, aminopeptidase N; Uhi, urban heat island

## Introduction

Pollination is a crucial ecosystem service, since >80% of flowering plant species—many of them providing food for humans (Klein *et al.,* 2007)—are pollinated by animals, especially insects (Ollerton *et al.,* 2011). However, there is increasing evidence of a decline in abundance, diversity and health of pollinators due to anthropogenic land use changes (Wagner *et al.,* 2021). Together with the expansion of agricultural land, urbanization is the main driver of human-induced land use change (Winfree *et al.,* 2009) that is currently affecting bees—the most important pollinators—both at intraspecific and community levels (Ferrari and Polidori, 2022; Geppert *et al.,* 2023; Polidori *et al.,* 2023; Gil-Tapetado *et al.,* 2024). Urbanization is often accompanied by the Urban Heat Island (UHI) effect, the rise in temperatures due to impervious (i.e. cemented) surfaces (Rizwan *et al.,* 2008). In addition, urbanization causes fragmentation and impoverishment of green areas (Li *et al.,* 2019), together with an anticipated plant phenology triggered by the UHI effect (Fisogni *et al.,* 2020). Furthermore, cities have higher levels of air and soil pollutants than surrounding areas (Baltensperger *et al.,* 2002; Morillo *et al.,* 2007).

Ultimately, such anthropogenic land use changes, whilst not necessarily negatively affecting bee diversity (Theodorou *et al.,* 2020), can impact bee morphology (Buchholz and Egerer, 2020; Tommasi *et al.,* 2022; Ferrari et al., 2024d and b), foraging behaviour and nutritional intakes (Biella *et al.,* 2022; Pioltelli *et al.,* 2024) or learning and memory (Leonard *et al.,* 2019; Démares *et al.,* 2022). These can have repercussions on bee health status, similarly to what is found in other invertebrates dwelling in urban habitats (Murray *et al.,* 2019). Such changes, in turn, can be used to provide a measure of the harmfulness or intensity of the anthropogenic disturbance (Buchholz *et al.,* 2020). Amongst the less explored phenotypic changes in bees due to urbanization pressure there are those related to morpho-physiology of the midgut, the part of the alimentary canal involved in digestion and nutrient absorption (Caccia *et al.,* 2019). Concerning other insects, it has been found both in wasps and in bees that urban-dwelling individuals had histological alterations in the midgut epithelium due to heavy metal contamination both in urban and agricultural areas (Polidori *et al.,* 2018; Ferrari et al., 2024a and d). Changes in physiological or biochemical traits, such as enzymatic activities in the midgut, have been largely unstudied in urban insects, although they can be used to assess the impact of anthropogenic pressures by revealing mechanisms that contribute to undermining insect health (Hyne and Maher, 2003; Leroy *et al.,* 2023).

Insect midgut is involved in the digestion and absorption of nutrients, with the former performed by both secreted and membrane-anchored enzymes (Caccia *et al.,* 2019). Responses associated with digestive enzymes can indicate stress conditions, and given that these enzymes influence mass, growth or mortality of the bee, they can be used in ecotoxicological studies (Vlahović *et al.,* 2012). Aminopeptidase N (hereafter, APN) is a proteolytic enzyme located in the brush border membranes of the insect midgut and capable of hydrolysing single amino acids from the N-terminus portion of the peptide chains (Adang, 2004). APNs are thus involved in the digestion of pollen, the main source of protein in the honeybee diet (Liolios *et al.,* 2015). In addition, proteolytic activity seems to correlate with the functional status of honeybees. Higher enzymatic activities are found in house bees (i.e. those performing in-hive tasks) than in foragers (i.e. those collecting nectar and pollen). This correlates with pollen consumption, as house bees consume more pollen, and therefore protein, than foragers (Moritz and Crailsheim, 1987).

Aminopeptidases have been used as indicators of stress or health status in insects, including bees. For example, worker honeybees infected with *Nosema* virus showed a reduction in their aminopeptidase activity (Matašin *et al.,* 2012). Moreover, Vlahović et al. (2014 and 2015) showed that larvae of a *Lymantria* species (Lepidoptera) artificially fed with cadmium had alterations in the aminopeptidase activity, suggesting that this parameter might be used as a biomarker to indicate the severity of gastrointestinal damage caused by cadmium ingestion. A decrease in proteolytic activity has also been shown in a *Musca* species (Diptera) in the presence of heavy metals (Blahovec *et al.,* 2006). In addition, the shredding of APN and alkaline phosphatase enzymes from brush border membranes into the midgut lumen has been described as a mechanism of response to ingested microbial pore-forming toxins in lepidopteran larvae (Valaitis, 2008; Hernández-Martínez *et al.,* 2010; Caccia *et al.,* 2012). Despite the potential importance of aminopeptidases as stress biomarkers in insects, no studies to date have linked urbanization pressure on insects to their digestive enzyme activity.

Here, we sampled honeybee (*Apis mellifera* Linnaeus 1758) foragers, the most widely managed pollinator that provides highly valued pollination services (Garibaldi *et al.,* 2013), along a gradient of anthropogenic land use change in the Metropolitan City of Milan (Northern Italy) and measured their APN activity. First, we briefly characterized the enzyme by testing its activity at different pH and temperatures to provide some functional information on this enzyme in *A. mellifera*. We then tested whether the UHI effect and land use characteristics found across the urbanization gradient influence the APN activity of the sampled bees. We hypothesized that bees sampled from homogeneous and impoverished urban green spaces, which are likely to host simplified floral communities (Biella *et al.,* 2022), should show a reduction in APN activity due to impoverished food resources and generally higher (thermal) stress due to urbanization. Our findings have substantial implications in guiding urban greenspace management to improve the environmental health of honeybees, and possibly other bee species.

## Materials and Methods

### Sampling activity

**Figure 1:**
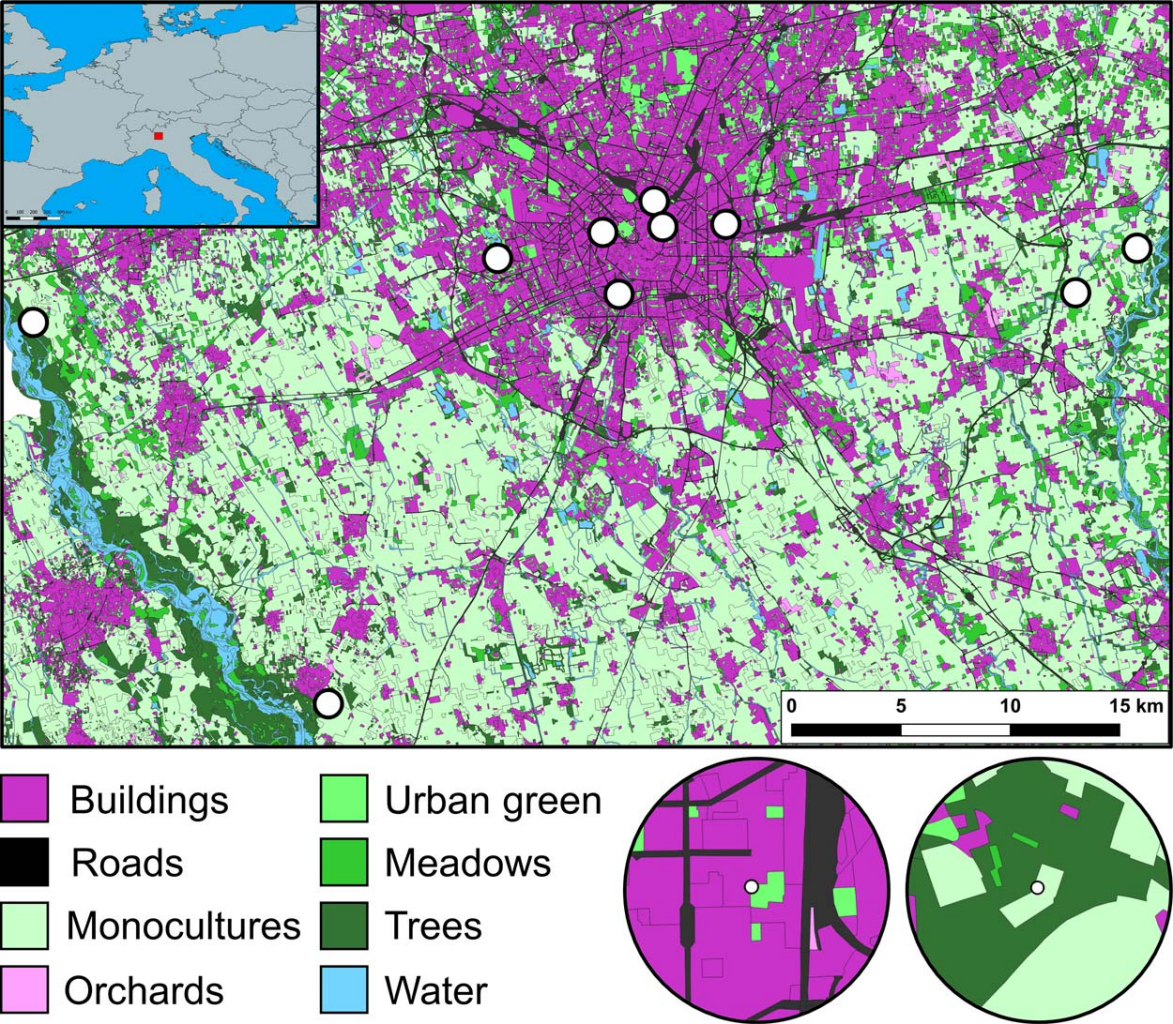
The sampling locations (dots) are plotted on a land use map of the Metropolitan City of Milan (the largest urban area in the centre of the map). The top-left panel shows the sampling location (square) in Europe. Below, two examples of land use around the sampling locations.

The study was performed in the Metropolitan City of Milan (centre of the study area: 45°28′01″ N; 9°11′24″ E), which includes the large city of Milan and the surrounding semi-natural and agricultural areas situated in Lombardy, Northern Italy. We selected a total of 10 sites (Fig. 1) with at least 1.5 km of separation along a land use gradient and sampled a total of 10 workers of *A. mellifera per* site between 1 June and 20 July 2023. To make the sampling as random as possible, it was performed at urban and semi-urban sites a few days apart. The insects were hand-netted on flowers, placed in plastic tubes with a foam rubber lid to allow the animal to breathe, and they were kept in cool bags to transport the bee back to the laboratory alive.

### Landscape characterization

For each site we extracted land use variables from non-overlapping 500-m buffers created from the shapefile provided by DUSAF6.0 (https://www.dati.lombardia.it/Territorio/Dusaf-6-0-Uso-del-suolo-2018/7rae-fng6), which contains a land use map of the entire Lombardy region with a resolution of 20 m. DUSAF6.0 contains >90 different land use categories, which we first summarized in the following four macro-categories: impervious areas (i.e. all cemented surfaces and man-made structures), green areas (i.e. all semi-natural covers), agricultural areas (i.e. all areas mainly used for the intensive cultivation of cereals) and water bodies. We then defined the landscape more finely using micro-categories derived from the categories listed above: buildings and roads derived from impervious areas, meadows (i.e. all flowered semi-natural grasslands), orchards, urban green areas (i.e. managed urban green areas) and trees derived from green areas. Since agricultural areas presented the same monoculture (wheat), we decided to include them in the same category. Overall, seven (excluding water bodies) land use categories were used in the data analysis (Fig. 1). For each site, we also calculated the Shannon diversity index (*H′*) of the land use micro-categories, a commonly used estimation of landscape heterogeneity (e.g. Martínez-Núñez *et al.,* 2023). Higher values of *H′* represent more heterogeneous landscapes.

For all the sampling sites, land surface temperature was estimated using the product MOD11A2 (https://modis.gsfc.nasa.gov/data/dataprod/mod11.php). For each bee, we assigned the value as the mean temperature of the month before the sampling recorded in the site. We expect this metric to represent the temperature conditions experienced by each bee during its life and foraging activities. Finally, for all the sites, the normalized difference vegetation index (NDVI, a vegetation productivity index) was retrieved through the product MOD13A1-061 (https://modis.gsfc.nasa.gov/data/dataprod/mod13.php). NDVI describes the amount of plant biomass in a place (e.g. Babar *et al.,* 2006). The higher its value, the higher the primary productivity of the green area is. As before, we assigned for each bee the mean NDVI recorded in the site 1 month previous its sampling.

All the raw data associated with landscape characterization are available in the Supporting information file Dataset.xlsx.

### Body size, midgut collection and aminopeptidase N activity

On arrival at the laboratory, the bees were anaesthetized on ice for 5–10 min. The midgut was then gently removed by pulling the stinger with forceps. Each midgut was then cut lengthwise to remove the peritrophic membrane with the enclosed content and Malpighian tubules. This operation was performed in a physiological solution of 1.8× phosphate buffered saline (PBS in distilled water to mimic the osmolarity of bee haemolymph of ~550 mosm/l) (Atmowidjojo *et al.,* 1999; Ali *et al.,* 2017). Each sample, stored in liquid nitrogen until use, was represented by the pool of two midguts. For each bee, we measured the intertegular distance (ITD, proxy for the body size) using a LEICA MZ75 stereomicroscope mounted with a LEICA Flexacam C3 camera (e.g. Ferrari *et al.,* 2024d).

For the enzymatic assays of APN, midguts were thawed and homogenized in ice-cold Hepes-Tris 10 mM pH = 7.2, 100 mM mannitol with an Eppendorf-fitting pestle in a 1.5-ml centrifuge tube. One μl of homogenization buffer *per* 1 mg of tissue was used. The homogenate was then filtered through a strainer (40-μm mesh size) fitting Eppendorf tubes by a quick spin at 10 000 × g at 4°C. The resulting suspension was blended and kept on ice until the assay.

To evaluate the urbanization effects, the activity of APN EC 3.4.11.2 was assayed using L-leucine p-nitroanilide (Sigma-Aldrich) as substrate (Bonelli *et al.,* 2019) and measuring its degradation by the release of p-nitroaniline (pNA). Different volumes of homogenate were diluted to 800 μl with 50 mM Tris–HCl, pH 7.5 and 200 ml of 20 mM L-leucine p-nitroanilide were added. Absorbance was measured continuously for 120 s at 410 nm at room temperature (20°C). One unit/mg (U/mg) of APN activity was defined as the amount of enzyme that releases 1 μmol of pNA per min per mg of proteins. Protein concentration of the homogenate was determined by the method of Bradford (1976), using bovine serum albumin as standard.

To briefly characterize APN functional properties in *A. mellifera* midgut, activity in midgut homogenates was evaluated at different pHs and temperatures. To measure the effect of pH, the same assay was performed using 50 mM Tris–HCl at pH 6, 7.5 and 9. To evaluate the effect of temperature on APN activity we performed the assay at the experimental temperatures of 4, 10, 20, 30, 40 and 50°C. This range covers the temperature excursion likely to be experienced by honeybees during our sampling period (20–40°C) as well as excessively cold and hot conditions. The APN activity was determined as described above, using the homogenate obtained from four midguts of bees collected in one of the sampling sites used (Site 03). The absence of spontaneous substrate hydrolysis under the different experimental conditions was checked performing the assays in the absence of homogenate. At least four technical replicates of activity measurement for each sample were performed.

### Statistical analysis

All the analyses were performed in the statistical software R (*v. 4.3.3,*  R Core Team, 2024). For the physiological characterization of the APN activity, the association between temperature and the enzymatic activity was tested using an ordinary linear model (OLM), whilst the effect of the three pH values were tested using an analysis of variance (ANOVA).

Then, how the landscape variables correlate with each other was explored both through a correlation matrix (Supplementary Fig. S1A) using Spearman’s ρ; and with a Principal Component Analysis (PCA) to visualize which variables were the most important in explaining differences between sites (Supplementary Fig. S1B). Urban and semi-natural sites formed two clear clusters, and from this visual separation only few variables *per* cluster were selected for the following statistical analyses due to high collinearity between them.

First, we excluded a possible effect of the sampling activity on the observed results using OLMs to test the effect of the sampling date on the APN activity. Then, we checked that different sampling sites had different values of APN activity with an ANOVA. Then, to analyse how the enzyme activity varied according to the land use gradient, Linear Mixed Models (LMMs) at two different scales were used. At the larger scale, the LMM included temperature, NDVI and landscape heterogeneity. This is a broad model that includes general aspects of the landscape. At a finer scale, the LMM included meadows, urban green parks and monocultures. This model allowed us to analyse specific land use characteristics such as those related to cities (urban parks), semi-natural areas (meadows) and the effect of agricultural practises. All LMMs included the sampling site as the random effect and the ITD as proxy for body size. With the package ‘performance’ (Lüdecke *et al.,* 2021) no deviances from residual and random effects normality were detected (Cheng *et al.,* 2010; Schielzeth *et al.,* 2020), and Variation Inflation Factors were always <4 (Harrison *et al.,* 2018). In the following section, results are presented as mean ± standard error. All the data used for the statistical analysis are reported in the supporting information file Dataset.xlsx.

## Results

The urbanization gradient was characterized by changes in land use and environmental parameters. In general, urban areas, with a higher proportion of impervious surfaces, were characterized by higher temperatures, fewer meadows (semi-natural flowered areas) and monocultures and larger managed urban green spaces (Supplementary Fig. S1A). Conversely, less urbanized areas were generally characterized by a higher proportion of meadows (if the area was semi-natural) or monocultures (if the area was agricultural) (Supplementary Fig. S1A). In addition, urbanization was inversely correlated with landscape homogeneity (Supplementary Fig. S1A).

**Figure 2:**
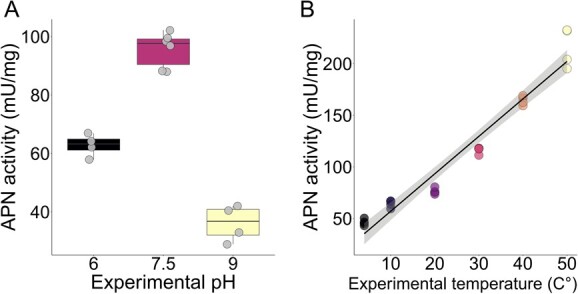
A) Boxplots showing the differences in the activity of APN at different pH conditions, points are actual values. B) Simple regression line (black) with 95% confidence interval (grey) showing the positive correlation between APN activity and the experimental temperatures. The points are actual values.

At room temperature (20°C), the variation in pH significantly affected the activity of APN in *A. mellifera* midgut (ANOVA: F*_2,11_* = 142, *P* < 0.001, Fig. 2A). APN activity was the highest at pH = 7.5, then pH = 6 and pH = 9. In addition, enzymatic activity increased with temperature (linear model: *P* < 0.001, R^2^ = 0.947, Fig. 2B) (Supplementary Table S3).

**Figure 3:**
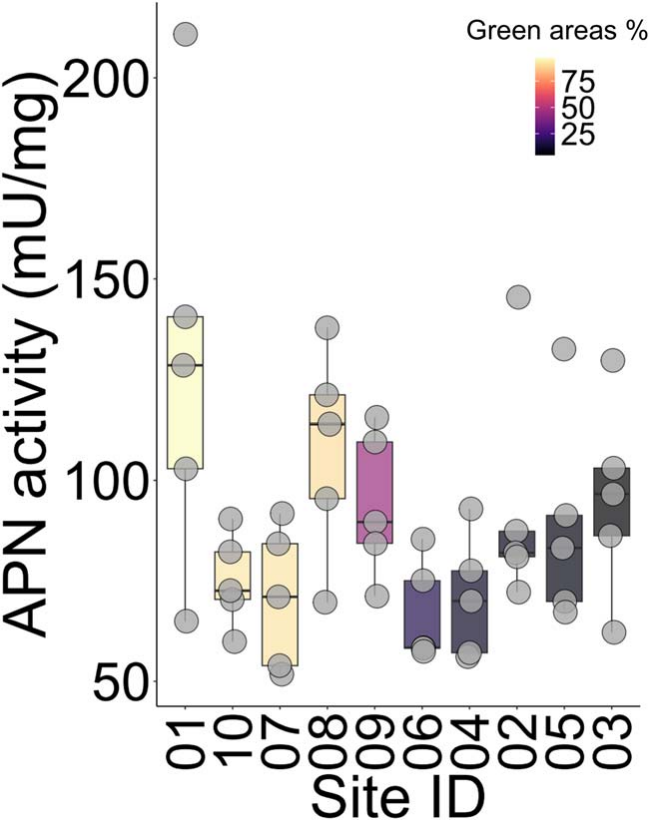
Descriptive boxplots showing the variation in the APN activity in the 10 sampling sites. Sampling sites are ordered based on the proportion of green areas (i.e. lower values indicate urbanized sites). Points are actual values.

APN activity in the bees collected from different sites varied between 51.744 mU/mg and 210.817 mU/mg (average 89.239 ± 11.239, Supplementary Table S2, Fig. 3). This was reflected by a statistically significant difference between sampling sites in terms of APN activity (ANOVA: F*_9,40_* = 2.754, *P* = 0.013), showing a significant intraspecific variability for this enzyme activity. The sampling date had no role in the observed variation of APN activity (ANOVA: *F_1,48_* = 2.467, *P* = 0.123).

**Figure 4:**
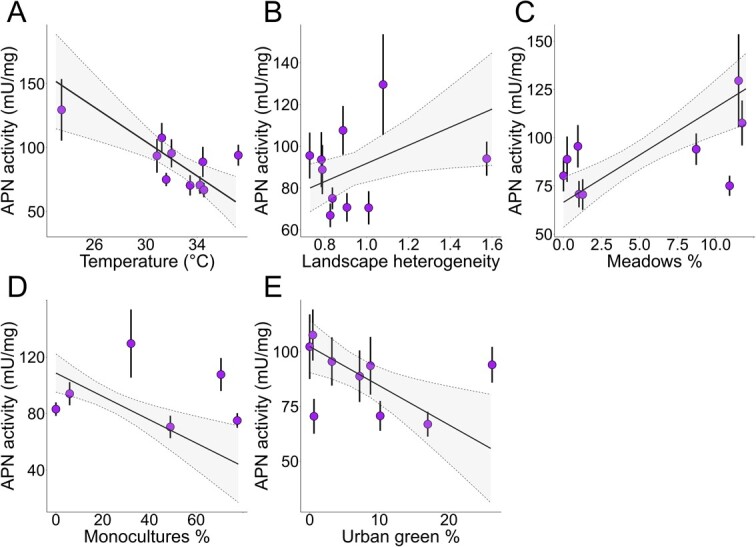
Scatterplots showing the statistically significant LMMs(P < 0.05). The points with ranges represent the mean and standard deviation of the variable in each site. The line represents the fitted model with the 95% confidence interval. In all the plots, the y-axis represents the APN activity plotted against A) mean site temperature, B) landscape heterogeneity (H'), C) proportion of meadows (semi-natural flowered areas), D) proportion of monoculture and E) proportion of urban (managed) green areas

APN activity varied across the anthropogenic land use gradient, both at large environmental scale and at small habitat scale (Table 1). Importantly, body size (ITD) was never statistically significant in explaining variations in APN activity, thus excluding possible allometric relationship between body size and APN activity. Using large-scale environmental variables, we found that the enzymatic activity was lower at warmer locations (Fig. 4A). In the field, temperature likely described the urbanization gradient, since it negatively correlates with the ratio between green and impervious surfaces (Pearson’s ρ = −0.759, *n* = 10, *P* = 0.011). We also found that APN activity was higher where the landscape was more heterogeneous (Fig. 4B).

**Table 1:** Summary of the LMMs performed

**Trait**	**d.f.**	**R**_**M**_ ^**2**^	**Predictors**	**Estimate**	**S.E.**	**Z**	***P***
APN (U/mg)	43	0.281	**Temperature (°C)**	−5.867	1.416	−4.142	**<0.001**
			NDVI	−62.656	34.036	−1.841	0.073
			**Landscape (*H′*)**	45.066	18.825	2.394	**0.021**
			ITD (mm)	−6.631	31.352	−0.211	0.834
	43	0.297	**Meadows (%)**	4.901	1.131	4.333	**<0.001**
			**Monocultures (%)**	−0.826	0.238	−3.474	**0.001**
			**Urban green (%)**	−1.788	0.626	−2.856	**0.007**
			ITD (mm)	−23.053	29.497	−0.782	0.439

APN: Aminopeptidase N, ITD: intertegular distance, NDVI: Normalized Difference Vegetation Index, S.E.: standard error, d.f.: degrees of freedom, R_M_^2^: marginal r-squared. Statistically significant results in bold (*P* < 0.05)

Using specific land use variables, we found that the enzymatic activity was higher in sites richer in meadows (Fig. 4C). The proportion of meadows has also to be considered as a proxy of urbanization since it is significantly and positively correlated with the ratio between green and impervious surfaces (Pearson’s ρ = 0.728, *n* = 10, *P* = 0.017). In addition, the APN activity was lower in sites richer in monocultures (Fig. 4D) and lower in sites with larger extension of urban parks (Fig. 4E). Despite there being no statistically significant correlation between the extension of urban parks and the green/impervious ratio (Pearson’s ρ = −0.601, *n* = 10, *P* = 0.066), the parks still represent urban environments that differ from more natural areas.

## Discussion

In this study, we first carried out a first brief characterization of the activity of midgut APN in *A. mellifera* (the most common bee species and pollinator in urban landscapes), and then we evaluated the enzymatic activity in field-collected workers along the urbanization gradient of a metropolitan city of northern Italy. Although the actual urbanization-related drivers remain unclear, we found evidence that this type of land use change may indeed affect the activity of this enzyme, with potential implications for honeybee health.

### Physiological characterization of APN activity in the laboratory

We found that the optimum of the APN activity was at pH = 7.5, in line with the pH present in *A. mellifera* midgut (Zheng *et al.,* 2017). This is in accordance with the activities of trypsin-like and chymotrypsin-like proteolytic enzymes of honeybees (Moritz and Crailsheim, 1987). Variations in APN activity due to changes in the pH were observed in other insects, with an optimal pH ranging from 7 to 8.5 (Parenti *et al.,* 1997; Adang, 2004; Cristofoletti *et al.,* 2006; Lomate and Hivrale, 2010). For example, in larvae of a *Helicoverpa* (Lepidoptera) species, the highest activity was recorded at pH = 7, with a slightly lower activity at pH = 6 and a much lower activity at pH = 9 (Lomate and Hivrale, 2010)*.* Similarly, in the larvae of the Lepidoptera *Bombyx mori* the maximum activity was reached at pH = 8 (Parenti *et al.,* 1997).

We also found a positive association of temperature (in the range 10–50°C) with APN activity. We decided to use 10–50°C as thermal limits since this is the temperature range in which honeybees are most likely active (Free and Spencer-Booth, 1959; Stabentheiner *et al.*, 2003 and 2007; Abou-Shaara *et al.,* 2012; Tomlinson *et al.,* 2015; Stupski and Schilder, 2021). This trend is consistent with findings in other insect species but constitutes the first evidence of this pattern in honeybees. For example, the optimal temperature for these enzymes was found to be around 50–60°C in a *Helicoverpa* sp.(Lepidoptera) (Lomate and Hivrale, 2010), 40°C in a *Morimus* sp. (Coleoptera) (Božić *et al.,* 2003) and between 37 and 42°C in an *Acanthoscelides* sp. (Coleoptera) (Osuala *et al.,* 1994). However, our data did not identify a peak in APN activity in the temperature range that we studied, suggesting that the optimum temperature *in vitro* is >50°C.

### Effect of urbanization on APN activity

We found that site temperature was the best predictor of APN activity. The highest activity was in the cooler (and most natural) site. In our case, temperature is the best environmental proxy for urbanization, as it correlates with the reduction of green areas in favour of cemented surfaces, thus describing the UHI effect (Deilami *et al.,* 2018). Since the sampling date, and therefore the seasonal increase in temperature, did not explain the variation in APN activity, we may exclude the possibility that our observations are the result of an acclimation effect. We may also exclude the possibility that temperature itself is the driver of reduced APN activity. In fact, the laboratory test allowed us to rule out the possibility that the actual site temperature (associated with urbanization) was the driver of what was observed in the field. In fact, we may hypothesize that the reduction in APN activity in warmer areas is possibly due to increased bee stress conditions in urban areas. Therefore, the effect we saw in the field-collected bees is most likely due to the altered or depleted environmental conditions of urbanized areas compared to the semi-natural sites; and not due to ambient temperature. This can also be supported by the fact that honeybees have efficient homeostatic mechanisms to buffer temperature fluctuations (Lima *et al.,* 2019).

This may be further supported by the fact that we would not expect a consistent pattern between acute exposure (laboratory experiments on homogenates) and chronic microclimatic temperatures (honeybees exposed to the UHI effect). In fact, although the laboratory experiment allowed us to perform a physiological characterization of these enzymes (a type of study that is still rare in the literature for *A. mellifera*), the exposure of the homogenates to the experimental temperatures does not necessarily correspond to the temperature found in the midgut of bees under field conditions. Hence, we can suggest that the enzyme activity has a higher thermal limit compared to the thermal limit of honeybee activity. This would demonstrate a considerable resilience of honeybees to temperature fluctuations, but more importantly it would highlight how urbanization is a multivariate agent that influences (often negatively) the physiology and health status of honeybees. We may ultimately hypothesize that in the field the mix of environmental variables that define urbanization likely interact to produce the reduction of APN activity in hotter sites we have found. Overall, this may support the hypotheses that a complex mix of urban habitat traits, and not the UHI effect *per se*, is the likely driver of the reduction in APN activity in honeybees.

Indeed, strong drivers of variation in APN activity were the proportion of meadows (semi-natural flowered areas) and the extent of urban parks (managed green areas), all correlated with the degree of urbanization. We found increased APN activity in sites richer in meadows, which are areas with great amount of natural vegetation. We can therefore hypothesize that honeybees that live in environments with greater pollen availability (semi-natural areas) may have gut enzyme profiles primed to digest more pollen and therefore a better metabolism (Di Pasquale *et al.,* 2013; Bryś *et al.,* 2021). We may also hypothesize that these flower-rich environments support healthier honeybee populations (Frias *et al.,* 2016) given their higher proteolytic activity. Indeed, it has been shown that, in bees, a diverse diet and the presence of flowering crops in semi-natural habitats (i.e. rich in meadows) can improve health, reproduction and ultimately resilience to stress (Vaudo *et al.,* 2015; Alaux *et al.,* 2017). Given the correlative nature of these results and the use of rough surrogates of food availability, future studies should more properly test for an association between the amount of protein in pollen and APN activity in this bee as well as in other bee species (Bataglia *et al.,* 2018). Since the enzymatic activity is substrate-inducible, we could expect to see higher enzymatic activity in bees feeding on high-protein pollen.

We also found that where urban managed parks are absent (i.e. in semi-natural areas) the enzymatic activity was high. Conversely, a decrease in APN activity was observed in sites with smaller parks (managed green areas) in a heavily urbanized matrix. This can be due to the fact that urban green areas are subject to intense mowing regimes that may jeopardize the availability of flower resources. Even though honeybees are generalist pollinators, a limited access to adequate flower resources (especially in a matrix such as a city) is a possible source of stress (Morimoto *et al.,* 2011; Branchiccela *et al.,* 2019). In addition, one of the highest APN activity values was observed in a very large suburban park in Milan. Again, this can be imputed to a possible beneficial effect of large green and flowered areas as buffers against urbanization (Ayers and Rehan, 2021).

Finally, landscape homogeneity and increase in the proportion of monocultures decreased the activity of APN, albeit in a less clear and pronounced manner. These are often flower-poor areas where most of the land is devoted to arable crops. However, this may be somewhat expected, given that simplification of the landscape has often substantial consequences for bees (Grab *et al.,* 2019). Indeed, these habitats are often accompanied by reduced floral availability (Decourtye *et al.,* 2010). In addition, the possible use of pesticides in highly agricultural areas may affect the proteolytic activity in the midgut of honeybees (Malone *et al.,* 1995; Burgess *et al.,* 1996; Wilde *et al.,* 2016). In addition, pollen-generalist bees—such as *A. mellifera*—have been shown to be positively affected by heterogeneous landscapes, where they are more likely to find their optimal nutritional niches (Parreño *et al.,* 2022).

### Conservation implications

Our work has important conservation implications. Given the importance of honeybees as urban pollinators (MacIvor *et al.,* 2017), it is crucial to understand the landscape elements that undermine their health status. Specifically, our results may suggest that the extent and quality of green spaces is a critical issue to address. Large urban parks (managed green areas), sites rich in meadows (semi-natural urban areas) and a heterogeneous landscape are most likely to maintain healthy honeybee populations in terms of APN activity. This is probably due to the quality of forage resources, as nectar and pollen nutrients have been shown to be affected by land use in urban areas (Pioltelli *et al.,* 2024). Bee conservation programmes in urban gardens may benefit from ensuring that rewarding plant species are present at high densities and/or spatially aggregated, possibly improving pollen richness/diversity and therefore forage quality (Biella *et al.,* 2022). However, more research is needed to better understand how to improve habitat quality in urban landscapes, as there is evidence that, whilst residential gardens can support native bees, remnants of urban bushland support an even wider range of species and are key to maintaining native bee populations (Prendergast *et al.,* 2022). Overall, enriching green spaces would potentially provide a healthier environment for bees.

### Conclusions

In conclusion, our results may suggest how the potential impoverishment of food sources due to urbanization, but not climatic shifts due to urbanization, could negatively affect APN activity in honeybees and possibly impair digestive functions (Terra and Ferreira, 2012).

In addition to those already discussed above, we also acknowledge some other limitations of our study, such as the non-negligible intraspecific variability and the fact that only foraging bees where sampled, whilst house bees or larvae from the hive were not considered. However, other works have often used foragers as bioindicators, since they are those most exposed to external agents. Despite such limitations, our results clearly evidenced a subtle but appreciable reduction in honeybee APN activity in highly anthropized areas, representing the first evidence of urbanization affecting a digestion-related physiological trait overall for insects.

Although further studies should be devoted to test the actual importance of each urbanization trait in order to assess their causal roles in bee physiology, this work emphasizes how better green management practises, such as reducing mowing practises to improve the quantity and quality of flower resources, can be an effective conservation method for bee fauna in urbanized areas.

Finally, our study opens up to new opportunities for research aimed at fully understanding the physiological and health issues caused by increasing anthropogenic land use changes in bees.

## Supplementary Material

Web_Material_coae073

## Data Availability

All raw data and other relevant information used to perform the statistical analysis are available in the Supporting information file Ferrari_et_al_Dataset.xlsx.
