# Supplementary material for: Urbanization-driven environmental shifts cause reduction in aminopeptidase N activity in the honeybee
Source: Conserv Physiol. 2024 Dec 12;12(1):coae073. doi: 10.1093/conphys/coae073 (PMC11636627; doi:10.1093/conphys/coae073)
Supplement: Web_Material_coae073 [file web_material_coae073.zip › Ferrari_et_al_Supporting_information_revised.pdf]

Supporting information for the ms:

Urbanisation-driven environmental shifts cause reduction in Aminopeptidase N activity in the honeybee

Andrea Ferrari[1], Silvia Caccia[2], Carlo Polidori[1]*

[1] Department of Environmental Science and Policy (ESP), University of Milan, via Celoria 26, 20133, Milan (Italy)

[2] Department of Biosciences, University of Milan, Milan, 20133, Italy

Table S1. Sampling sites with geographical coordinates, mean temperature and Normalised Difference Vegetation Index (NDVI).

Site	Latitude	Longitude	Temperature (°C)	NDVI
01	45.435017	8.829432	23.440	0.811
02	45.472099	9.162463	30.868	0.446
03	45.475301	9.234414	32.001	0.382
04	45.446619	9.171834	34.215	0.521
05	45.484892	9.19252	34.441	0.443
06	45.474412	9.197582	34.527	0.425
07	45.278069	9.001908	33.448	0.802
08	45.464731	9.474934	31.245	0.619
09	45.461487	9.100893	37.201	0.513
10	45.4465	9.438861	31.587	0.690

Table S2. Sampling sites with mean land use variables used in the statistical analyses.

Site	APN (U/mg)	Green %	Impervious %	Buildings %	Meadows %	Monocoltures %	Orchards %	Roads %	Trees %	Urban green %	Water %	Landscape heterogeneity	Green / Impervious	Agricultural / Impervious
01	129.543 ± 21.553	96.636	3.364	3.364	11.516	32.111	0	0	53.009	0	0	1.079	28.726	9.545
02	93.583 ± 11.791	8.725	91.275	71.442	0	0	0	19.833	0	8.725	0	0.784	0.096	0
03	95.548 ± 9.845	4.447	95.553	75.357	0.944	0	0.316	20.196	0	3.187	0	0.728	0.047	0
04	70.720 ± 6.114	11.137	86.647	71.452	1.014	0	0	15.195	0	10.122	2.216	0.906	0.129	0
05	88.826 ± 10.535	7.400	92.600	71.318	0.234	0	0	21.282	0	7.166	0	0.788	0.080	0
06	66.911 ± 5.070	16.963	83.037	69.997	0	0	0	13.040	0	16.963	0	0.826	0.204	0
07	70.523 ± 7.118	91.838	8.162	8.162	1.258	48.921	0	0	41.049	0.609	0	1.010	11.251	5.993
08	107.618 ± 10.434	90.424	9.576	9.576	11.746	70.589	0	0	7.648	0.442	0	0.887	9.443	7.371
09	94.051 ± 7.314	44.553	44.730	42.133	8.727	5.882	1.667	2.596	2.070	26.207	10.717	1.574	0.996	0.132
10	75.069 ± 4.670	92.047	4.282	4.282	10.926	77.566	2.811	0	0.744	0	3.671	0.834	21.495	18.113

Table S3. Physiological characterisation of Aminipeptidase N (APN) activity at different pH and temperature conditions. Values are reported as mean ± standard error. The samples come from the sampling site #03.

**Factor**	**Value**	**APN (U/mg)**
pH	6	62.823 ± 1.905
	7.5	95.542 ± 2.447
	9	36.118 ± 3.082
Temperature	4°C	46.306 ± 1.532
	10°C	64.185 ± 1.755
	20°C	76.386 ± 1.497
	30°C	116.202 ± 1.617
	40°C	164.390 ± 2.139
	50°C	215.920 ± 9.577

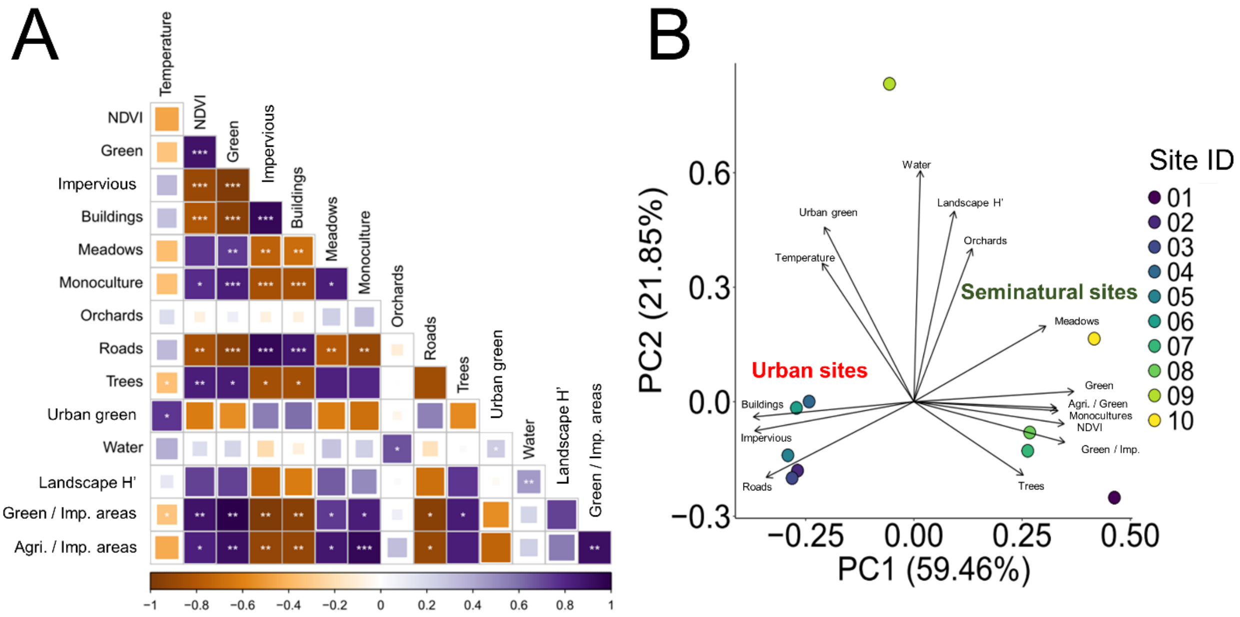

Figure S1. A) correlation matrix (Spearman's ρ) of the environmental variables used to describe the sampling sites. Asterisks indicate statistically significant correlations. B) Visualisation of the Principal Component Analysis (PCA) used to visually cluster the sampling sites based on the landscape characteristics. NDVI: Normalised Difference Vegetation Index.
