# Supplementary figures and images for: Urbanization-driven environmental shifts cause reduction in aminopeptidase N activity in the honeybee
Source: Conserv Physiol. 2024 Dec 12;12(1):coae073. doi: 10.1093/conphys/coae073 (PMC11636627; doi:10.1093/conphys/coae073)

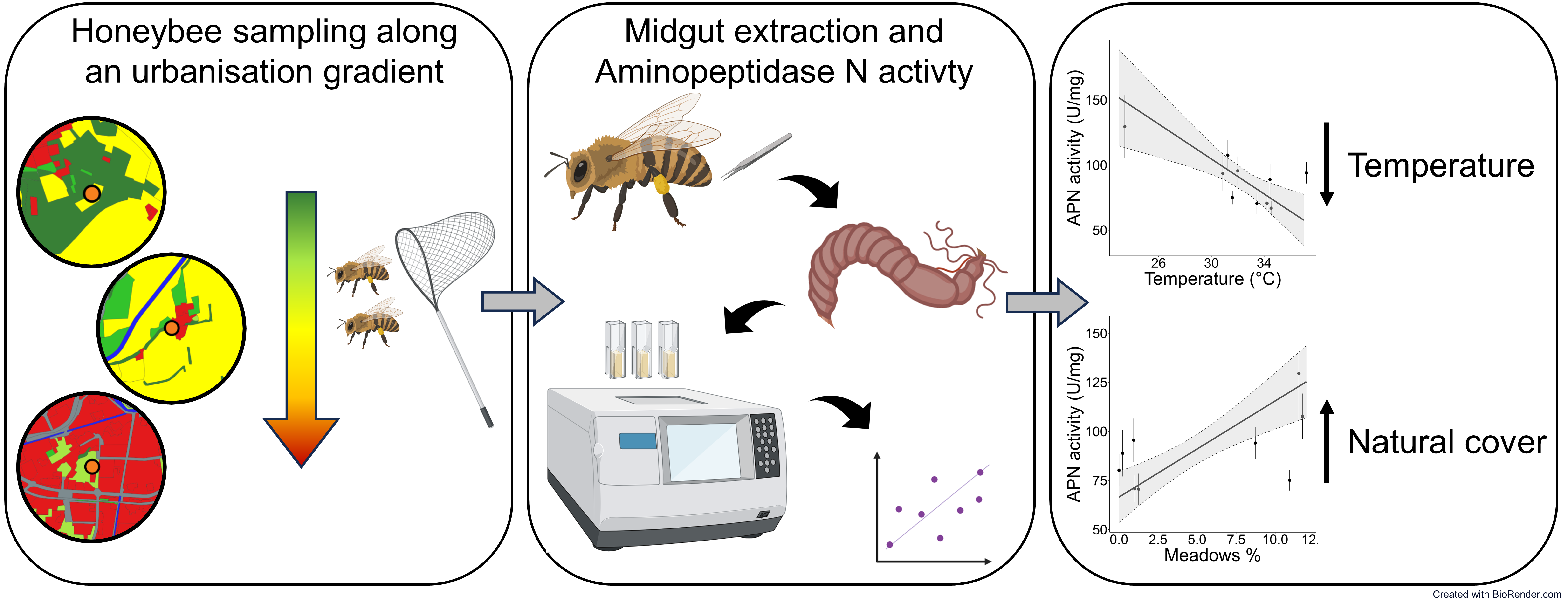

Supplement: Web_Material_coae073 [file web_material_coae073.zip › Ferrari_et_al_GraphicalAbstract.TIF]
